# Longitudinal Invariance of the Strengths and Difficulties Questionnaire Across Ages 4 to 16 in the ALSPAC Sample

**DOI:** 10.1177/10731911221128948

**Published:** 2022-10-18

**Authors:** Lydia Gabriela Speyer, Bonnie Auyeung, Aja Louise Murray

**Affiliations:** 1 University of Cambridge, UK; 2 The University of Edinburgh, UK

**Keywords:** longitudinal measurement invariance, confirmatory factor analysis, Strengths and Difficulties Questionnaire, ALSPAC

## Abstract

The Strengths and Difficulties Questionnaire (SDQ) has been widely used to study children’s psychosocial development longitudinally; however, such analyses assume longitudinal measurement invariance, that is, they presuppose that symptom manifestations are measured comparably across different ages. Violations of this assumption could bias longitudinal analyses and should therefore be empirically tested. This study tested longitudinal measurement invariance within a confirmatory factor analysis framework in the U.K.-based Avon Longitudinal Study of Parents and Children (*N* = 13,988). Results indicated that SDQ scores showed configural, metric, scalar, and residual invariance across ages 7, 8, 9, 11, 13, and 16, supporting its use for comparing variances, covariances, and means over time within a latent variable model as well as using observed scores. At age 4, configural invariance was not supported, indicating that mental health symptoms as measured by the SDQ manifest differently at this age, thus necessitating caution when comparing symptoms as measured by SDQ scores at this age to later ages.

The Strengths and Difficulties Questionnaire (SDQ) is one of the most widely used omnibus measures of children’s psychosocial development (Goodman, 1997). It measures emotional and behavioral development in five domains: conduct problems, emotional problems, peer problems, hyperactivity/inattention, and prosocial behaviors and has been translated into nearly 80 languages (Kersten et al., 2016). The SDQ has been used in a variety of settings, including in clinical and educational practice where it has been used to inform clinical services and to evaluate the effectiveness of interventions (Sosu & Schmidt, 2017). The SDQ has also been a measure of choice in longitudinal studies tracking children’s mental health over development (Anderson et al., 2007; Connelly & Platt, 2014; Hirota et al., 2022; O’Neill et al., 2019; Wang et al., 2021). For instance, its scores have been used to examine predictors of heterogeneity in hyperactivity/inattentive behaviors over development (Murray, Hall, et al., 2021), to investigate the effect of video game playing on the development of peer problems and prosocial behaviors during late childhood and early adolescence (Lobel et al., 2019) and to gain insights into the developmental relations between emotional and conduct problems over childhood and adolescence (Speyer et al., 2022).

While the psychometric properties of the SDQ have been extensively evaluated, generally finding favorable results (Kersten et al., 2016), less attention has been paid to evaluating whether the SDQ measures symptoms in a comparable manner across different developmental stages. Considering that childhood and adolescence are characterized by a range of marked social, cognitive, physical, and biological changes (Eccles, 1999; Rapee et al., 2019), it is not only possible but highly likely that symptoms manifest differently at different developmental stages. For example, various types of anxiety are more or less relevant at different stages. Whereas attachment anxiety tends to be more prominent during childhood (Madigan et al., 2013), social anxiety tends to increase during adolescence in conjunction with a heightened sensitivity for social reward (Caouette & Guyer, 2014). Conduct problems also change over development with physical forms of aggression being relatively normative in early childhood, but an indicator of more severe difficulties when manifesting during adolescence (Côté et al., 2006). In addition to changing symptom manifestations, the context in which behaviors occur also changes, for instance from occurring in the home context in which they can be readily observed by parents, to occurring in the school context in which teachers are likely to have better insights (De Los Reyes et al., 2015). With regards to the psychometric qualities of the SDQ, these developmental and contextual changes may manifest as a violation of longitudinal measurement invariance over time. Longitudinal measurement invariance analysis is used to assess whether the same constructs, such as the domains of psychosocial development in the SDQ, are measured comparably at different time points within the same sample (Edwards & Wirth, 2009; Liu et al., 2017). This helps ensure that any changes in observed scores are due to true changes over time, rather than to differences in what the SDQ measures at each time point, for instance if symptoms manifest differently at age 7 compared to age 13.

Depending on the inference that researchers seek to draw from longitudinal data, different levels of invariance are required. These can be tested within a Confirmatory Factor Analysis (CFA) framework in which latent factors (i.e., constructs such as conduct problems) are defined using multiple observed variables (i.e., questionnaire items) (Millsap & Yun-Tein, 2004). Factor loadings represent the strength of the association between each item and the underlying factor while, for ordinal items, thresholds are additionally modeled to capture the value of the latent factor at which an individual transitions from being in one category to being in the next category (e.g., transitioning from “somewhat agree” to “agree”) (Millsap & Yun-Tein, 2004). The most basic level of invariance is configural invariance which requires the same number of factors as well as the same pattern of factor loadings across each time point (Liu et al., 2017). In the case of the SDQ, the theoretically hypothesized pattern of factor loadings is a 5-factor model in which items from each SDQ subscale load onto one latent factor (prosocial behaviors, peer problems, hyperactivity/inattention, conduct problems, emotional problems), with latent factors allowed to covary (Goodman, 1997). In the psychometric literature, at least 12 different factor structures have been proposed in addition to the hypothesized 5-factor model (Gomez & Stavropoulos, 2019), most notably including a 3-factor model including an internalizing, externalizing and prosociality factor (Croft et al., 2015). However, of these proposed factor structures, the 5-factor model has been most widely supported by factor analytic work and has tended to show the best fit also when directly comparing it to other factor models, including the 3-factor model (de la Cruz et al., 2018; Gomez & Stavropoulos, 2019; Kersten et al., 2016; Sosu & Schmidt, 2017). If it can be established that the pattern of factor loadings does indeed follow the same hypothesized 5-factor structure across time, the next step is to test for metric invariance. Metric invariance is required if one is interested in comparing regression paths or variances and/or covariances over time within a Confirmatory Factor Analytic (CFA) framework. This may, for example, be necessary when the interest is in whether conduct problems have the same effect on academic achievement in primary versus secondary school. Metric invariance requires factor loadings to be equal across time but allows for differences in the thresholds of ordinal items over time (Liu et al., 2017; Liu & West, 2018). When interested in comparing factor means (construct levels) over time, for instance, to examine the trajectory of hyperactivity/inattention across childhood and adolescence, scalar invariance is required. In this case, both factor loadings and thresholds are required to be equal across time (Liu et al., 2017; Liu & West, 2018). If, rather than using latent factors, observed scores are compared over time, residual invariance also needs to hold, requiring, for ordinal items, equal residual variances in addition to equal factor loadings and thresholds across time (Liu et al., 2017).

To date, only a handful of studies have investigated longitudinal measurement invariance of the SDQ. Croft et al. (2015) examined the longitudinal invariance of the parent-reported SDQ in the U.K. population-representative *Millennium Cohort Study* across ages 3, 5, and 7, finding support for metric and scalar invariance for the hyperactivity/inattention, conduct problems and prosocial behavior subscales; but only support for metric invariance for the emotional and peer problems scales. Sosu and Schmidt (2017) tested longitudinal invariance across ages 4, 5, and 6 in the *Growing up in Scotland* study, finding support for configural, scalar, and metric invariance across all subscales. Most notably, Murray, Speyer, et al. (2021) extended Croft et al.’s (2015) study, testing longitudinal invariance for the SDQ in the MCS across a much wider age range (ages 3–17) finding support for configural, metric, and scalar invariance across ages 5, 7, 11 and 14, but not for ages 3 and 17 at which time points configural invariance did not hold, suggesting that developmental differences at these time points may partly reflect differences in measurement.

While these findings are encouraging, suggesting that the SDQ generally generates scores suitable for tracking children’s psychosocial development across time, it is important to establish measurement invariance in each new sample as measurement invariance does not necessarily translate from one sample to another. One of the most widely used longitudinal cohort studies that included the SDQ as a measure of children’s psychosocial development is the Avon Longitudinal Study of Parents and Children (ALSPAC) (Boyd et al., 2013; Fraser et al., 2013). ALSPAC is a U.K.-based birth cohort study that has been following the lives of around 14,000 children born between 1991 and 1992 up until today. At the following median ages, 4, 7, 8, 9, 11, 13, and 16, ALSPAC collected parent-reported SDQs which have been widely used to investigate research questions relating to children’s psychosocial development (Barker & Maughan, 2009; Bauer et al., 2021; Dachew et al., 2021; Fetene et al., 2020; Howe et al., 2013; Pourcain et al., 2011; Riglin et al., 2016; Speyer et al., 2021; Tejerina-Arreal et al., 2020). However, it has not yet been investigated whether longitudinal invariance analyses would indeed support SDQ scores as longitudinal measures of children’s mental health development in the ALSPAC sample. This is an important gap in the literature as a lack of invariance testing leaves open the possibility that studies making inferences on longitudinal development based on the SDQ in the ALSAPC sample may be biased by differences in measurement. Furthermore, while invariance analyses should ideally form a basic component of any longitudinal analysis, not all researchers are necessarily equipped to conduct such analyses. To reduce concerns that the measure being used violates assumptions such as longitudinal invariance, researchers consequently rely on existing literature to have comprehensively evaluated the scales they are using. Such comprehensive evaluations should include replications of findings in independent samples, as establishing longitudinal invariance in different samples would considerably strengthen the evidence base for using the SDQ in existing as well as new longitudinal studies. Thus, in this study, we aim to replicate prior findings suggesting that the SDQ provides scores that reliably and comparably capture different domains of psychosocial functioning across different developmental stages by conducting a longitudinal measurement invariance analysis in the ALSPAC sample (ages 4, 7, 8, 9, 11, 13, and 16).

## Methods

### Participants

Participants were part of the Avon Longitudinal Study of Parents and Children (ALSPAC), a longitudinal birth cohort study. Pregnant women resident in Avon, United Kingdom, with expected dates of delivery April 1, 1991, to December 31, 1992, were invited to take part in the study. The initial number of pregnancies enrolled is 14,541 (for these at least one questionnaire has been returned or a “Children in Focus” clinic had been attended by 19/07/99). Of these initial pregnancies, there was a total of 14,676 fetuses, resulting in 14,062 live births and 13,988 children who were alive at 1 year of age (Boyd et al., 2013; Fraser et al., 2013). When the oldest children were approximately 7 years of age, an attempt was made to bolster the initial sample with eligible cases who had failed to join the study originally, resulting in an additional 913 children being enrolled. Thus, the total sample size for analyses using any data collected after the age of 7 is 15,454 pregnancies, resulting in 15,589 fetuses. Of these, 14,901 were alive at 1 year of age. The study website contains details of available data through a fully searchable data dictionary and variable search tool (http://www.bristol.ac.uk/alspac/researchers/our-data/).

### Ethical Considerations

Ethical approval for the study was obtained from the ALSPAC Ethics and Law Committee and the Local Research Ethics Committees. Informed consent for the use of data collected via questionnaires and clinics was obtained from participants following the recommendations of the ALSPAC Ethics and Law Committee at the time. For further information, see: http://www.bristol.ac.uk/alspac/researchers/research-ethics.

### Measures

The parent-reported SDQ measures children’s psychosocial development in five domains: emotional problems, peer problems, conduct problems, hyperactivity/inattention, and prosocial behavior. Each subscale is made up of five items scored on a 3-point Likert-type scale (*not true, sometimes true*, and *certainly true*) with an additional response option for *can’t say/not applicable.* The full list of SDQ items and the subscale they correspond to is provided in Table 1. As mentioned in the introduction, the psychometric properties of the SDQ have been widely evaluated with most studies supporting the structural and convergent validity of the 5-factor model of the SDQ (for a review, see Kersten et al., 2016). While internal consistency values of SDQ scores have sometimes been suggested to be lower than desired, in the current sample, omega values (McDonald, 1999) suggested good internal consistency. In particular, we observed the following omega values for conduct problems: .72, .77, .82, .81, .84, .84, .85; for emotional problems: .73, .78, .81, .82, .82, .82, .85; for hyperactivity/inattention: .83, .84, .87, .84, .85, .84, .84; for prosocial behavior: .81, .84, .86, .82, .83, .84, .85 and for peer problems: .63, .76, .76, .79, .81, .81, .79; thus, only at age 4, internal consistency for peer problems were lower than the conventionally accepted threshold for acceptable reliability of .70. Omega values were calculated using poly-choric correlations to account for the fact that response options were ordered-categorical (Loomans et al., 2014). In the ALSPAC sample, the SDQ was completed by parents when children were median aged 4 (*N =* 9519), 7 (*N =* 8457), 8 (*N =* 7851), 9 (*N =* 8109), 11 (*N =* 7394), 13 (*N =* 7086), and 16 (*N =* 5693).

**Table 1.** SDQ Items.

Subscale	Item
Conduct problems	Often having temper tantrums
Conduct problems	Generally being obedient
Conduct problems	Often fighting with or bullying other children
Conduct problems	Often lying or cheating
Conduct problems	Stealing from home, school, or elsewhere.
Hyperactivity/inattention	Being restless, overactive, being unable to stay still for long
Hyperactivity/inattention	Constantly fidgeting or squirming
Hyperactivity/inattention	Being easily distracted
Hyperactivity/inattention	Thinking before acting
Hyperactivity/inattention	seeing tasks through to their end
Emotional problems	Often complaining of headaches, stomach-aches or sickness
Emotional problems	Having many worries
Emotional problems	Being often unhappy, down-hearted, or tearful
Emotional problems	Being nervous or clingy in new situations
Emotional problems	Having many fears, being easily scared
Peer problems	Being rather solitary and tending to play alone
Peer problems	Having at least one good friend
Peer problems	Generally liked by other children
Peer problems	Being picked on or bullied by other children
Peer problems	And getting on better with adults than other children
Prosocial behaviors	Being considerate of other people's feelings
Prosocial behaviors	Sharing readily with other children
Prosocial behaviors	Being helpful if someone is hurt
Prosocial behaviors	Being kind to younger children
Prosocial behaviors	Often volunteering to help others

*Note.* All items were scored on a three-point Likert-type scale (*not true, sometimes true, certainly true*). SDQ = Strengths and Difficulties Questionnaire.

### Statistical Analysis

Longitudinal invariance was tested within a CFA Framework, (for details see Liu et al., 2017). We chose to use a CFA approach rather than another framework such as Item Response Theory (IRT) as CFA approaches more directly focus on testing measurement equivalence on the scale rather than on the item level (D’Urso et al., 2021). In addition, CFA is more commonly used for investigating the psychometric properties of the SDQ. This means that findings from a CFA approach are most easily accessible to researchers interested in using the SDQ. Models were fit in Mplus 8.7 (Muthén & Muthén, 2018) using the Mplus “convenience features” for invariance testing using delta parameterisation for configural, metric and scalar invariance testing and theta parameterisation for residual invariance testing (see Version 7.1 Mplus Language Addendum available at https://www.statmodel.com/download/Version7.1xLanguage.pdf for details). Specifically, we first fitted a configural model based on the theoretically implied 5-factor structure of the SDQ. For the configural model, the pattern of factor loadings were fixed to be the same at each time point; however, the magnitude of loadings and thresholds were allowed to vary between the individual time points. For model identification, the first factor loading for each factor was fixed to one at each timepoint and factor means fixed to zero. If configural invariance was supported, we proceeded to test for metric invariance by introducing cross-time point equality constraints for factor loadings, i.e., factor loadings for each item were forced to be equal at each time point. To achieve model identification, means were set to zero at the first time point and the first threshold of each item held equal across all time points. If metric invariance could be achieved, scalar invariance was tested by additionally fixing all item thresholds to be equal at each time point. Finally, if scalar invariance was supported, we added residual invariance constraints in a model using theta parameterisation to test for residual invariance by fixing all residual variances to 1 and comparing this to a scalar invariance model in which only the residual variances for the first group are fixed to 1 while all other residual variances are freely estimated (Brown, 2015). Model fit was judged using the following model fit indices: comparative fit index (CFI) >.90, Tucker–Lewis Index (TLI) >.90, root mean squared error of approximation (RMSEA) <.06, and standardized root mean square residual (SRMR) <.08 (Hu & Bentler, 1999). To judge whether metric invariance held, we followed the guidelines proposed by Chen (2007). While many different guidelines for judging invariance have been proposed (some more and others less conservative, e.g., Finch & French, 2018; Rutkowski & Svetina, 2017), we selected the criteria proposed by Chen (2007) as we considered them to be well-suited to identifying non-trivial measurement invariance violations in longitudinal models. Following Chen (2007), metric invariance would be supported if, compared to the model fit of the baseline model, decreases in CFI were below .010, increases in RMSEA were below .015 and increases in SRMR were below .030. The same criteria (except using increases of .010 as cut-off for SRMR) were then used to judge if scalar and residual invariance held, comparing the model fit of a metric invariant model to a scalar invariant model and a scalar invariant model to a model with residual invariance constraints imposed (Chen, 2007). If changes in model fit suggested that metric or scalar invariance could not be established, we examined modification indices as well as expected parameter changes and iteratively released constraints to explore whether a partially metric or scalar invariant model could be found. Such a partially invariant model may be sufficient to compare (co)variances or means over time, provided that noninvariance is appropriately modeled within a latent measurement model (Edwards & Wirth, 2009; Pokropek et al., 2019).

If configural invariance was not supported for the full longitudinal model, we proceeded to examine single-group models at each time point to first investigate whether configural noninvariance may be due to a lack of model fit at a specific time point. If this was the case, we would proceed to test for configural invariance across selected time points only (as well as metric and scalar invariance if configural invariance was supported). If single-group models suggested that model fit was not acceptable at most time points, we concluded that longitudinal measurement invariance was not supported on the configural level across development and consequently did not test for metric and scalar invariance. All models were estimated in Mplus 8.7 (Muthén & Muthén, 2018) using weighted least squares means and variance (WLSMV) estimation to account for the ordered-categorical response format of the SDQ items. In WLSMV estimation as implemented in Mplus, missing data are handled using pairwise deletion, thus all observed data are used to produce parameter estimates. This approach yields consistent parameter estimates under the assumption that data are missing completely at random (Asparouhov & Muthén, 2010).

## Results

The configural model showed poor fit according to CFI (.884), TLI (.869), and RMSEA (.061) but acceptable fit according to SRMR (.073), for full model results, including factor loadings and threshold parameters, see the Open Science Framework (OSF): https://osf.io/35bfd/?view_only=b96345c69dde4e3cb3cfaca95629c91f. As such, we examined single-group models at each time point to identify potential sources of configural noninvariance. Table 2 lists the model fit indices for all single-group CFAs including links to the full model output provided on OSF. Results suggested that model fit was poor at all ages according to TLI and CFI, but acceptable at all ages except age 4 according to RMSEA and SRMR. Interestingly, prior psychometric evaluations of the SDQ have often found similar results in that a 5-factor model of the SDQ was supported based on RMSEA and SRMR but not based on TLI and CFI (Gomez & Stavropoulos, 2019; Kersten et al., 2016). Following this observation, Gomez and Stavropoulos suggested that CFI and TLI are likely to be too conservative as a model fit index for the SDQ as they are based on comparing a theoretical model to a null model, that is a model with zero correlations between all variables. CFI and TLI values will consequently be low if the theoretical model has low correlations among the variables (Kenny, 2020), which Gomez and Stavropoulos suggested to be the case for the SDQ. Consequently, CFI and TLI may not be the ideal indicator of model fit for the SDQ; instead, Gomez and Stavropoulos advocated using RMSEA and SRMR as the primary indicators of model fit of the SDQ (Gomez & Stavropoulos, 2019). Such recommendations are also in line with recent research on model fit indices that has suggested that model fit should not be judged based on general cut-offs but should take individual characteristics of the data such as sample size, number of items and number of factors into account (McNeish & Wolf, 2021).

**Table 2.** Model Fit Indices for Single-Group Models.

Age	CFI	TLI	RMSEA	SRMR	Link to full output
4	.845	.824	.067	.083	https://osf.io/kwruz/
7	.888	.873	.058	.068	https://osf.io/yb8f9/
8	.891	.876	.066	.073	https://osf.io/k6nxd/
9	.891	.877	.055	.066	https://osf.io/7qzwe/
11	.900	.887	.056	.067	https://osf.io/y84je/
13	.891	.877	.060	.071	https://osf.io/afv64/
16	.891	.877	.060	.079	https://osf.io/42y7n/

*Note.* CFI = Comparative Fit Index; TLI = Tucker–Lewis Index; RMSEA = root mean square error of approximation; SRMR = standardized root mean square residual.

Although CFI and TLI did not support the 5-factor structure of the SDQ at individual time points, we tested again for configural invariance in a longitudinal model but only included ages 7 to 16, the timepoints for which configural invariance was supported according to RMSEA and SRMR. Results of this model were in line with the single-group models, suggesting that configural invariance held according to RMSEA (.059) and SRMR (.070), but not according to CFI (.892) and TLI (.878). We subsequently proceeded with introducing metric invariance constraints leading to a deterioration in model fit for CFI and SRMR (CFI = .890; TLI = .883; RMSEA = .058; SRMR = .072); however, the changes in model fit were within the limits specified by Chen (2007), thus supporting metric invariance. We proceeded to test for scalar invariance, finding that model fit again deteriorated according to CFI and SRMR (CFI = .887; TLI = .887; RMSEA = .057; SRMR = .073) but the decreases in model fit were within the limits suggested by Chen (2007), hence, indicating that scalar invariance can be achieved. We consequently also tested for residual invariance, finding that model fit improved (CFI = .890; TLI = .897; RMSEA = .055; SRMR = .073), thus we concluded that residual invariance held across ages 7 to 16 in the ALSPAC sample. Model fit indices and differences between models for CFI, RMSEA, and SRMR are listed in Table 3.

**Table 3.** Model Fit for Longitudinal Invariance Models.

Model	CFI	ΔCFI	RMSEA	ΔRMSEA	SRMR	ΔSRMR	Link to full output
Configural: 4–16	.884	—	.061	—	.073	—	https://osf.io/wfs36/
Configural: 7–16	.892	—	.059	—	.070	—	https://osf.io/63nj4/
Metric: 7–16	.890	.002	.058	−.001	.072	.002	https://osf.io/63nj4/
Scalar: 7–16	.887	−.003	.057	−.001	.073	.001	https://osf.io/63nj4/
Residual: 7–16	.890	.003	.055	−.002	.073	.000	https://osf.io/vpsg3

*Note.* CFI = Comparative Fit Index; TLI = Tucker–Lewis Index; RMSEA = root mean square error of approximation; SRMR = standardized root mean square residual; To judge whether metric or configural invariance held, we followed the guidelines proposed by Chen (2007). Metric invariance was supported if, compared to the model fit of the baseline model, decreases in CFI were below .010, increases in RMSEA were below .015 and increases in SRMR were below .030. The same criteria (except using increases of .010 as cut-off for SRMR) were used to judge if scalar and residual invariance held, comparing the model fit of a metric invariant model to a scalar invariant model and a scalar invariant model to a model with residual invariance constraints imposed.

## Discussion

The goal of this study was to evaluate longitudinal measurement invariance of a popular measure of child psychosocial development, the SDQ, in a widely used longitudinal study, the ALSPAC cohort. Longitudinal invariance is an implicit assumption in a majority of longitudinal analyses from simple analyses such as *t*-tests to more complex analyses such as growth mixture modeling or random intercept cross-lagged panel modeling. Yet the assumption of longitudinal invariance is seldom tested. We found that the fit of the SDQ was poor at age 4 but adequate at ages 7 to 16. Scalar invariance could be achieved across the age range of 7 to 16, supporting the position that provided scores are modeled across time within a latent variable model, unbiased estimates of change in the constructs over time are attainable. Furthermore, we also found support for residual invariance, suggesting that observed SDQ scores can be used to study changes in SDQ constructs over time. Overall, the findings of our study add important evidence suggesting that the SDQ is a suitable instrument for studying psychosocial development in young people aged 7 to 16.

Given that we found support for residual invariance, results support the testing of a variety of different longitudinal research questions spanning the age range of 7 to 16 using the ALSPAC cohort. Without ensuring that metric, scalar, and residual invariance holds, such analyses may be biased in a difficult-to-predict direction. Any observed changes in covariances or means could be reflective of changes in their measurement over time rather than reflective of true changes that may help illuminate the mechanisms underlying mental health problems. Current results suggest that, if modeled within a latent variable model as well as using observed scores, the SDQ can indeed be used to study the development of co-occurring mental health issues, for instance using graphical vector autoregression or random-intercept cross-lagged panel models which rely on comparing covariances over time (Russell et al., 2018; Speyer et al., 2022). Furthermore, the SDQ can also be used to examine changes in (latent) means over time, for example, allowing for examination of trajectories of mental health problems in the ALSPAC cohort, as well as predictors and outcomes of individual differences in trajectories (Barker & Maughan, 2009; Speyer et al., 2021).

Findings of this study are broadly in line with previous longitudinal invariance analyses of the SDQ. For example, prior analyses of developmental invariance in the Millennium Cohort Study, a different U.K.-based birth cohort, have suggested that the SDQ shows invariance across ages 5 to 14 up to the partial residual level, suggesting its suitability for comparing means, variances, and covariances over development (Murray, Speyer, et al., 2021). Interestingly, Murray et al.’s study further found that the SDQ did not show configural invariance at age 3 and here we observed configural noninvariance at age 4. Taken together, this suggests that symptoms measured by the SDQ manifest differently in the earliest ages for which it is designed. One reason for this observation may be that the manifestation of mental health problems are less differentiated during early childhood, that is they are less specific to a particular type of problem behavior but indicate a more general propensity to mental health problems (Murray et al., 2016). This has been supported by a number of studies, finding that mental health symptoms become more differentiated over development (Cole et al., 1998; Lahey et al., 2004). If symptoms were indeed less differentiated in early childhood, this would suggest that symptoms hypothesized to be indicative of one type of problem behavior (e.g., conduct problems) could also be indicative of a different type of problem behavior (e.g., emotional problems). Examining modification indices and expected parameter changes indicated that the inclusion of a number of cross-loadings, for instance, a cross-loading of the hyperactivity/inattention item *can stop and think before acting* on the prosociality factor, may help improve model fit, thus supporting the notion that mental health symptoms may not be as differentiated during the toddler years. Another potential reason for the lack of invariance at ages 3 and 4 may relate to the fact that some of the mental health issues measured in the SDQ tend to not have an onset this early in life or may be more difficult to observe. For instance, emotional problems and in particular anxiety have a median onset of age 11 (Kessler et al., 2005) whereas hyperactivity/inattentive behaviors have an earlier onset, but often only get detected once children transition to school (Cherkasova et al., 2013). Recently, the SDQ has been used as early as age 2 (D’Souza et al., 2019). Findings here highlight that SDQ scores for 2 year olds need to be carefully checked for their comparability to older age groups if they are going to be used for comparing symptoms at different stages of development.

Overall, the level of invariance achieved in the current sample was good. This suggests that SDQ items are appropriately generic to identify problematic behaviors that are independent of specific developmental stage during the age range of 7 to 16. The disadvantage of this, however, is that the SDQ is not well calibrated to capture developmentally specific manifestations of mental health problems. For instance, the SDQ is likely not suited to capture manifestations of anxiety that may be more prominent at certain developmental age ranges, such as social anxiety during adolescence or attachment anxiety in childhood (Caouette & Guyer, 2014; Madigan et al., 2013). Similarly, the SDQ is unlikely to appropriately capture changes in manifestations of aggression. While aggression may be more of a reactive nature during childhood, in conjunction with increasing cognitive control abilities and heightened importance of peer relations, proactive, and in particular social forms of aggressive behaviors become more common during adolescence (Girard et al., 2019). Considering that such changes in symptom manifestations cannot be captured by a measure designed to assess mental health problems across a wide age range, it is important to complement studies investigating changes in mental health symptoms using the SDQ with studies that use more specific measures designed to capture age-specific manifestations of mental health problems.

While this study has a number of strengths, including the large sample size and the availability of the SDQ for a large age range spanning both childhood and adolescence, a number of limitations also need to be considered. First, even though the included time points span a large age range, there are gaps in some ages, particularly during early childhood and in late adolescence. For instance, we were not able to examine whether the lack of invariance is limited to age 4 and younger (as observed in Murray, Speyer et al., 2021) or may extend to ages 5 and 6. Similarly, we were not able to examine whether the SDQ would show longitudinal invariance during later adolescence. The only study to date that investigated longitudinal invariance for a longer time-span than this study (Murray, Speyer et al., 2021) suggested that configural invariance of the SDQ was not supported at age 17 in the Millennium Cohort Study (MCS). Thus, it remains to be confirmed whether this more generally holds true for the SDQ or whether this is specific to the MCS. Future longitudinal studies are needed to address these gaps. Second, invariance testing is inherently subjective. Not only is there considerable choice in modeling approaches that allow for invariance testing, including Item Response Theory, Exploratory Structural Equation Modeling and CFA approaches, the interpretation of fit cut-offs used to determine invariance varies substantially between studies depending on what magnitude of noninvariance is judged to be substantively problematic. This is particularly challenging in the context of testing invariance for measures based on ordinal-categorical items as much less research to date has investigated how invariance should be judged in this context. Simulation studies on model fit have suggested that optimal fit cut-offs depend to a large extend on the population model, that is the number of items, factors, and groups, as well as on the sample characteristics (e.g., sample size) (Sharma et al., 2005). With regards to the interpretation of configural model fit of the SDQ, prior research has already suggested that traditional fit indices may not be suitable to judge model fit as these consistently give conflicting information (Gomez & Stavropoulos, 2019; Kersten et al., 2016). Gomez and Stavropoulos suggested that CFI and TLI are likely too conservative as they are based on comparing a theoretical model to a null model with questionnaires based on theoretical models that assume low correlations between variables performing poor on these indices. However, this has not yet been investigated rigorously using simulation studies, thus future methodological research is needed to confirm this suggestions and to derive more objective indicators of measurement invariance more generally. Finally, analyses of attrition in the ALSPAC cohort have suggested that children with more behavioral difficulties are more likely to drop-out (Wolke et al., 2009). This may have impacted invariance analyses in a hard-to-predict direction. However, using a simulation study, Wolke et al, also found that the nonrandom attrition observed in ALSPAC only marginally affected the validity of analyses conducted in the cohort, thus, effects of missing data on measurement invariance are likely to be minimal.

Results of this study, suggesting a lack of invariance between age 4 and other ages, underline the importance of testing for longitudinal measurement invariance of scores from developmental mental health measures. Future studies should extend this work by examining longitudinal invariance in other popular measures of child and adolescent mental health. To date, invariance is not commonly tested, however, this should become a necessary prerequisite to the testing of any model that relies on comparing means, variances, or covariances (or statistics derived from them) over time. Future studies should also consider longitudinal invariance during the development of new measures. This could help ensure that at least the required number of items that are necessary to achieve partial metric invariance of scores are included as core questionnaire items while additionally allowing for increased insights into potential differences in interpretation of items at different ages (Edwards & Wirth, 2009; Sass, 2011).

## Conclusion

In conclusion, the SDQ as measured across ages 7, 8, 9, 13, and 16 in the ALSPAC cohort shows longitudinal invariance up to the residual level. This supports its use for the testing of developmental hypotheses that involve comparing means, variances, and covariances over time, both using observed scores as well as within a latent variable model. Caution is needed when comparing SDQ scores at later ages to SDQ scores at age 4 as configural invariance was not supported at this time point. This indicates that mental health difficulties measured by the SDQ may manifest differently in early childhood.
